# Observations on the Modus Operandi of Opium

**Published:** 1802-11-01

**Authors:** 

**Affiliations:** Manchester


### *Observations on the Modus Operandi of Opium; communicated by* Mr. Ward, *of Manchester.*

[ Continued from Vol. VIII. p. 325—348. ]

BEFORE I take my leave of the Inquiry, I have a few remarks to make upon that part of the concluding chapter, which treats of the ufe or abufe of opium in particular difeafes.

In entering upon this part of the work, it is impoffible to avoid being ftruck with a moft important defect in Doctor Crumpe's theory; and which is fufficient of itfelf, to prove the juftice of the fentence lately paffed upon it; namely, *that it is utterly incapable of affifting us in directing, either when, or how, to adminifter opium, or of elucidating the effects refulting from it; nor can any one regulate his profeffional conduct by it, without forfeiting all claim to confiftency; becaufe a facrifice muft unavoidably be made, either of theory to practice, or of practice to theory. For inftance, if Dr. Crumpe be correct in maintaining, that opium acts folely as a ftimulant,* p. 168—170, 179, 192, &c. &c. *he is highly culpable, firft, for having recommended it in difeafes where ftimulants are inadmiffible,* (as, where want of fleep precedes or accompanies delirium, (p. 234); in diarrhœa, whether it occur as a primary difeafe, or as a fymptom of debility in fever, (p. 234—236, 295); in cholera, (p. 294); in the latter ftages of pneumonia and pleuritis, when the urgent fymptom is a cough, proving the chief caufe of the continuance of pain, and want of fleep, (p. 256—7); in phthifis, where a troublefome cough and diarrhœa occur, (p. 280); to alleviate irritation, and diminifh the fecretion of bile in cholic, (p. 293) &c. &c.); *and fecondly, for having neglected to recommend it in difeafes where ftimulants are particularly indicated*; as in fyncope; arthrodynia; paralyfis of long ftanding, where there are no evident marks of compreffion, dyfpepfia, amenorrhœa, chlorofis, fcrophula, fcorbutus, rachitis, &c. &c.

It is true that he has recommended it in various difeafes where debility has fupervened; as in the laft ftage of fever, &c. &c. and attributes its tonic effects to its ftimulant properties; though it is demonftrable that they proceed, *in all the cafes in which Dr. C. has prefcribed it, from its diminifhing the irritability and mobility either of the whole fyftem, or of particular parts, i. e. from a directly fedative operation*; and I believe I may with confidence appeal to experience when I affert, that *in no cafe whatever has it the effect of ftrengthening the fyftem, but where, in confequence of its directly fedative properties, it either procures fleep, allays fpafm, or eafes pain.*

Several inftances are alfo related in this chapter, where fuch effects refulted from the exhibition of opium, as plainly demonftrate the futility of Dr. Crumpe's arguments.

" *Whatever foundations there may be for thefe fpeculations,* (fays Dr. C.) *it is certain, that opium has been frequently exhibited to advantage,* ALLAYING THE FURY OF THE ENRAGED, AND CALMING THE AGITATION OF THE IRRITABLE." p. 298.

In page 279, we are told *that a large dofe of opium was* IMMEDIATELY

MEDIATELY *effectual in a very violent case of epistaxis, which had resisted every other remedy, external and internal.*

Again, "Beryat recommends the exhibition of an opiate an hour before the expected commencement of the paroxysm, especially if the cold stage be vehement and attended with considerable tremor and rigour; observing at the same time, that if given sooner it will frequently fail; if during the hot stage, that it may prove dangerous. *His fears, however, on this head, are groundless*; for Dr. Lind has found, that opiates given *half an hour after the formation of the hot stage*, both rendered the remaining part of the paroxysm milder, produced a more perfect intermission, and rendered a less quantity of bark necessary to the completion of the cure." p. 244.

It would be tedious to enumerate all the instances of inconsistency which occûr in the course of the chapter; they may be met with in almost every page; and were opium to be given in such diseases only as are taken notice of by Dr. Crumpe, its usefulness would be confined within very narrow bounds.

I ought before to have noticed the circumstances alluded to in the following passage, as Dr. C. seems to have thought them peculiarly favorable to his theory.

"Striking, however, as are the various proofs already given of the stimulant qualities of opium, another circumstance remains to be mentioned equally forcible and convincing, which is, that when custom has rendered its employment absolutely necessary to some constitutions, if any accident prevents those in so unfortunate a situation from obtaining a sufficient supply, the bad consequences resulting from such deficiency can only be obviated by the frequent and liberal use of other powerful stimulants. Of this fact a striking instance is related by Acosta, and thus transcribed by Dr. Alston in the Edinburgh Medical Essays: "There were," says he, "some Turkish prisoners and Arabian captives in the ship in which I returned from the Indies to Portugal, who had a small quantity of opium concealed, and used it only as a medicine. When they had consumed it all, one of them, a Turk of Aden, said to me, Since you have the care of the sick, I must let you know, that unless you give me and my companions opium, we cannot live two days. I denied I had any. The only remedy then, said the Turk, whereby we who have been accustomed to eat opium can be recovered, is by a draught of pure wine every morning; though this is very hard and uneasy to us, as being contrary to our law, yet since our health depends upon it, we must submit. By his advice I gave them all wine; they recovered, and in a month's time would take no more wine, and neither needed nor desired opium." Inq. p. 177, 178.

It

It is evident that in this inftance, the wine proved both a fubftitute and an antidote, which proves too much; for had its modus operandi been exactly fimilar to that of opium, *the relief would have been only temporary*; *there muft, therefore, have been some effential difference in the properties of the two articles*, which it may be worth while to inveftigate.

From Dr. Crumpe's analyfis of opium, and his experiments upon the effects of its different component principles,* it appears,

1. "That opium is compofed of a gum, a refin, an effential falt, and of earthy indiffoluble impurities.

2. "The quantity of gum and refin is nearly equal; the proportion of the falt very inconfiderable; the earthy impurities amount to three parts out of twelve.

3. "The gum, when *perfectly* feparated from the refin, is divefted of the peculiar properties of opium, poffeffes no degree of aftringency, but retains the whole of the bitternefs of the medicine.

4. "The refinous matter is void of bitternefs, but poffeffes as well the whole of the aftringency of the medicine as of the peculiar and narcotic properties for which it is celebrated.

5. "The fmall portion of effential falt which opium contains, is analogous to that of other vegetable fubftances, and poffeffed of no peculiar properties.

6. "Whether it be occafioned by the prefence of the faline matter, or by the attraction between the gum and refin, the union of both is fo ftrong, that the refin cannot be perfectly feparated from the gum by the action of different menftrua.

7. "Any fuch feparation of the component parts of the medicine is of no ufe whatever in medical practice." Inquiry, p. 85—87.

Confequently Dr. Crumpe is warranted in afferting, (fee p. 167) that opium cannot be feparated into any two principles endowed folely with the oppofite qualities of a ftimulant and fedative. It will therefore be allowable, *when speaking of its action*, to confider it as a fimple fubftance; but the fame remark will not apply to wine. We know from its analyfis, that it may be feparated into two or more principles endowed with oppofite properties.

All

---

* I confider this chapter (the third) as the only valuable part of the Inquiry, and am happy in bearing teftimony to the merit of Dr. Crumpe, for the great attention he paid to the fubjects of it.

All wines contain alcohol, (the quantity of which is in proportion to their ftrength and purity*) fugar, and acid of tartar.† It is allowed on all hands, that alcohol operates in the fame manner as opium; *and I fhall now confider it as an eftablished fact, that the modus operandi of the latter, and of course of the former, is directly sedative.*‡ The nutritious and ftimulant qualities of fugar,§ and the tonic and ftimulant properties of the acid of tartar,‖ are alfo generally known and acknowledged. Thefe fubftances therefore, together with the aromamatic principle, where that is prefent, will greatly counteract the fedative effects of the alcohol; *and to this cause is the recovery of Acofta's patients to be attributed.* Hence we learn, that *pure wine* may prove an excellent fubftitute for opium, *where the latter is taken with a view to its exhilirating effects:* it is alfo extremely well calculated, *if the quantity be diminifhed, and the intervals protracted*, to remove the bad effects arifing from its immoderate ufe, and may be reforted to with advantage by thofe who are defirous of difcontinuing the practice; but for this purpofe, its efficacy may be increafed by boiling the wine, and infufing in it fome *aromatic stimulant*, as cinnamon or cloves, fugar

---

* Cullen's Mat. Med. vol. i. p. 410, 413; vol. ii. p. 315. Nicholfon's Chem. Dict. Art. Wine, and Spirit, ardent.

† "It appears from this imperfect analyfis, that wine confifts of water, ardent fpirit, colouring matter of a refinous nature, fugar, tartar, and tartareous acid, and an aromatic principle." Nicholfon's Ch. Dict. vol. ii. p. 867.

‡ Farther proofs may be thought neceffary, and thefe I fhall endeavour to fupply in a fucceeding paper.

§ "The crop time in the fugar iflands is the feafon of feftivity, both to man and beaft; for fo agreeable to the tafte, and fo nourifhing to the corporeal frame, is the juice of the cane, that every animal derives health and vigour from its ufe. Such of the negroes as were meagre and fickly, become furprifingly altered for the better in a few weeks after the mill is fet in action. The labouring horfes, oxen, and mules, though almoft conftantly at work during this feafon, yet, in confequence of eating plentifully of the green tops of this invigorating plant, and being indulged with fome of the fcummings from the boiling-houfe, improve more than at any other period of the year. Even pigs and poultry fatten on the refufe. In fhort, during crop time, plenty and induftrious chearfulnefs every where prevail in fuch a high degreee on a well regulated plantation, as confiderably to foften the hardfhips of flavery, and induce an impartial fpectator to conclude that the miferies of life are fometimes exaggerated through the delufive medium of fancy."

Edwards's Hiftory of the Weft Indies, Vol. ii. p. 226—7. See alfo Hunter on the Venereal Difeafe, p. 354—6, and Cullen's M. M. v. i. p. 229—232; v. ii. p. 402—7, &c. &c.

‖ "The fame acids (vegetable acids) are never in fuch a concentrated ftate as to fhew any cauftic or even ftimulant powers; but they fhew readily the ftimulant power which is in the weaker or much diluted acids, fo far as they excite appetite and promote digeftion; and probably it is by the fame power that they excite the urinary excretion." Cullen's M. M. v. ii. p. 332, 512—13.

fugar being added fo as to make it palatable; and this procefs fhould never be omitted where there is any fufpicion of its being adulterated.

"Profper Alpinus," continues Dr. C. "during his long refidence among the Egyptians, remarked that many who accuftomed themfelves to chew opium conftantly, if but deprived of it for a fingle day, became languid, dejected, and uneafy, at the cuftomary hours of taking it, and could only be roufed from this ftate by the ufual quantity of opium, or by a large draught of wine rendered ftill more powerful by the addition of pepper and other aromatics." "Animi fiquidem, fays he, deliquio faftidiofiffimo tentantur nulloque auxilio fic tuto liberantur, quam rurfus opium devorantes. Multos ab hac fervitute liberatos vidi, fi, in hora qua foliti fint ipfum capere, largius ex vino Cretico, pipere, atque aliis aromatibus alterato potent."* Inq. p. 178—179.

In this, therefore, as in almoft every other inftance throughout the Inquiry, it appears that the conclufions to be drawn from the facts Dr. Crumpe has adduced, inftead of confirming his arguments, have a diametrically oppofite tendency. Happy would it be were this the only producible inftance, of a man, in general well informed, perfifting in the maintenance and defence of opinions, demonftrably erroneous. Dr. Crump's language on the prefent occafion is by no means warrantable.

"The various circumftances I have mentioned, as evincing the exiftence of a ftimulant quality in opium, will even, if confidered fingly, be found to contain very ftrong proof of the truth of the affertion; but taken in the aggregate, their force is fo ftriking, that I know no medical queftion on which we can decide with any certainty, if not on the prefent." Inq. p. 179.

I truft, however, that every impartial perfon who has thought the fubject deferving of attention, is by this time convinced, that there is not the leaft foundation for fuppofing opium to act as a ftimulant.

To expofe the fallacy of this deftructive doctrine was the object of the firft part of this paper; and I indulge a hope that the facts and arguments I have brought forward, will be found fo clear, fatisfactory, and decifive, as to remove all uncertainty with regard to this moft momentous queftion.

Some of my expreffions in animadverting upon the Inquiry may poffibly be thought fevere; but I am not confcious of their being more fo than the occafions required, or freedom of difcuffion permits. I have never ftarted an objection without giving either reafons, or proofs, or both; nor have I, knowingly,

* De Medicina Egyptiorum, lib. 4. cap. i.

omitted omitted to notice any of the facts or arguments which seemed favourable to Dr. Crump's opinions, and to be deserving of attention.

I shall only delay the pleasure I anticipate in taking leave of the Inquiry, while I observe, that I do not imagine a parallel instance can be produced of any publication on a scientific subject having gained an equal degree of credit, on such flimsy pretensions.

*Manchester, Oct.* 8, 1802.

[ To be continued. ]

